# Hippocampal subfield morphology from first episodes of bipolar disorder type II and major depressive disorder in a drug naïve Chinese cohort

**DOI:** 10.3389/fpsyt.2024.1438144

**Published:** 2024-07-25

**Authors:** Enzhao Cong, Yingyan Zhong, Mengyue Wu, Haiying Chen, Yiyun Cai, Zheng Ling, Yun Wang, Hui Wen, Yao Hu, Huifeng Zhang, Yan Li, Xiaohua Liu, Pingfang Zhong, Weijie Lai, Yifeng Xu, Yan Wu

**Affiliations:** ^1^ Shanghai Mental Health Center, Shanghai Jiao Tong University School of Medicine, Shanghai, China; ^2^ Shanghai Tenth People’s Hospital, School of Medicine, Tongji University, Shanghai, China; ^3^ X-LANCE Lab, Department of Computer Science and Engineering, MoE Key Lab of Artificial Intelligence, AI Institute Shanghai Jiao Tong University, Shanghai, China; ^4^ Affective Disorder Department, Lincang Psychiatric Hospital, Lincang, China; ^5^ Psychiatric Department, Zhangzhou Fukang Hospital, Zhangzhou, China

**Keywords:** BD-II, MDD, hippocampus, subregions, magnetic resonance imaging

## Abstract

**Introduction:**

Symptoms during the onset of major depressive disorder [MDD] and bipolar disorder type II [BD-II] are similar. The difference of hippocampus subregion could be a biological marker to distinguish MDD from BD-II.

**Methods:**

We recruited 61 drug-naïve patients with a first-episode MDD and BD-II episode and 30 healthy controls (HC) to participate in a magnetic resonance imaging [MRI] study. We built a general linear model (one-way analysis of covariance) with 22 hippocampal subfields and two total hippocampal volumes as dependent variables, and the diagnosis of MDD, BD-II, and HC as independent variables. We performed pair-wise comparisons of hippocampal subfield volumes between MDD and HC, BD-II and MDD, BD-II and HC with *post hoc* for primary analysis.

**Results:**

We identified three regions that differed significantly in size between patients and controls. The left hippocampal fissure, the hippocampal–amygdaloid transition area (HATA), and the right subiculum body were all significantly larger in patients with MDD compared with the HC. In the onset of first-episode of MDD, the hippocampal volume increased significantly, especially on the left side comparing to HC. However, we found differences between MDD and BD-II were not statistically significant. The volume of the left HATA and right subiculum body in BD-II was larger.

**Conclusions:**

The sample size of this study is relatively small, as it is a cross-sectional comparative study. In both MDD and BD-II groups, the volume of more left subregions appeared to increase. The left subregions were severely injured in the development of depressive disorder.

## 1 Introduction

The characteristics of depressive episodes of bipolar disorder type II [BD-II] are very similar to those of a major depressive disorder [MDD]. However the prevalence of BD-II is 4.5% less than that of MDD (16.2%) (1) and easily misdiagnosed in early episodes (2). The difficulty in identifying the first episode of depression in BD-II increases the possibility of misdiagnosis (2, 3). This misdiagnosis affects the choice of treatment options, and BD-II depressive episodes are often accompanied by anxiety characteristics and a high risk of self-injury and suicide (4, 5). If a depressive episode of BD-II is misdiagnosed as a depressive episode of MDD and then they were prescribed antidepressants, it can lead to mixed episodes or manic episodes, increasing the risk of suicide (6, 7). Therefore, early identification of depressive episodes of BD-II and intervention are very important. Exploration of the biological markers of BD-II lays the foundation for early diagnosis and intervention (8). Being able to distinguish BD-II from MDD early in the course of the disease would allow the provision of appropriate and effective treatment (9, 10). In this paper we set out to find biomarkers that would distinguish BD-II from MDD.

We chose to examine the hippocampus because of earlier findings suggesting that the size of the hippocampus might alter with changes in mood, and that cellular and molecular mechanisms associated with mood disorders were localised to specific hippocampal subfields (11). The hippocampus has important functions in the regulation of emotion and declarative memory (12). It has been shown that the volume of the hippocampus is smaller in MDD (13–18) and also smaller in bipolar disorder (19, 20). However, few studies have compared hippocampal substructures in MDD with BD-II depression (21, 22). A series of articles including Cao et al., and an ENIGMA Consortium study found that hippocampal volume was significantly reduced and changed in patients with bipolar disorder type I [BD-I] (11, 23, 24) or in a bipolar disorder affected group including BD-I and BD-II (25). However, there are few articles exploring specifically BD-II depressive episodes which are similar to episodes of MDD. Exploring the differences between these is very important and leads to an exploration of important markers for differentiation.

Some researchers found that hippocampal subfield volume reductions were more prominent in patients with MDD than with BD-II (26), while others found patients with BD-II had reduced volumes of the hippocampal subfields compared with those with MDD, especially in the left CA4, GCL, ML and both sides of the hippocampal tail (11)). Furthermore, the duration of bipolar disorder was negatively correlated with the volume of the hippocampal subfields, which evidenced the neuroprogressive nature of BD-II (24, 26). The specific reduction of the hippocampal subfield in MDD is found in the cornu ammonis and dentate gyrus (27). The differences of hippocampal subfields between MDD and BD-II are helpful in understanding hippocampal neuroplasticity in them (27) and in discriminating them through structural MRI data (28).

Some researchers believe that BD-II is a progressive neurodegenerative change (Schneider, DelBello et al., 2012; Abe, Ching et al., 2022) and bipolar disorder progresses at the same time as the volume of the hippocampus shrinks (Cao, Bauer et al., 2016 (29). For BD-II, the CA1 area in the hippocampus is believed to be reduced, which may be an important sign of severe mental disorder (30). However, in these studies, the fact that patients were undergoing treatment and the severity of the disorder were not considered, and the recurrence of the disorder and the specific type of BD were often regarded as unimportant factors, with notably few studies exploring the impact of the early development stage of BD-II on the hippocampus.

In this paper we hypothesize that: 1). We hypothesize that patients with BD-II will have same changes in the volume of left hippocampus as in patients with MDD comparing to controls. 2) There may be differences in the brain structure of patients with BD-II compared with patients with MDD. 3) Specifically, we asked whether for BD-II there may be less dominant reduction in some subregions of the hippocampus, such as cornu ammonis 1 [CA1] or granule cell-molecular layer-dentate gyrus [GC-ML-DG].

## 2 Methods

### 2.1 Participants

A total of 30 patients with the first episode of BD-II depression (18-60 years old), and 28 patients with the first episode of MDD (18-60 years old) were recruited from the Shanghai Mental Health Center, in Shanghai, in the People’s Republic of China between January and December 2021. Using the patient edition of the Structured Clinical Interview for DSM-IV Axis I Disorders (SCID-I/P) patients were evaluated to see whether they met the diagnostic criteria for of BD-II and MDD(Those patients with BD-II currently have moderate or severe depressive symptoms. When reviewing their medical history, they have had mild manic episodes and were diagnosed with bipolar disorder. At the time of enrollment, the patients were still experiencing depressive symptoms.). Before the patients were further evaluated, their clinical symptoms were assessed according to the 24-item Hamilton Depression Rating Scale [often abbreviated to HRSD, HDRS or Ham-D) (31) and Hypomania Checklist [HCL-32) (32) but only for BD-II. The diagnosis was reviewed by an attending psychiatrist and deputy chief-psychiatrist to confirm that the diagnosis was consistent. For bipolar disorder, only patients with BD-II depression were enrolled. Inclusion criteria: age 18-60 years, right hand-dominant, meeting DSM-IV diagnosis criteria for MDD or BD-II; and drug-naïve patients with first-episode depression; for MDD patients, a total HDRS score of >20, and for BD-II, an HCL-32 score of >13 and an HDRS score of >20. Exclusion criteria: 1) Patient history of another DSM-IV Axis I disorder (e.g. schizophrenia, schizo-affective disorder or mental retardation). 2) Serious or unstable physical diseases such as tumours or cardiovascular disease, alcohol/substance abuse or any other severe physical disease. 3) Primary neurological diseases such as vascular disease or cognitive impairment. 4) Contraindications for MRI scanning including metal implants, dental braces or fear of claustrophobia. 5) Being in receipt of medication or physical therapy before enrolment. HC were age-matched and their HDRS score was checked to ensure that it was < 20 at the time recruitment advertisements were put up in the community by the study doctor co-ordinating the case group. HCs needed to meet the following criteria: 1) 18-60 years old. 2) Met the criteria of the non-patient edition of the Structured Clinical Interview for DSM-IV Axis Disorders (SCID-NP). 3) They were not suffering from any current or past physical disease. 4) They had no family history of psychiatric illness. Everyone who participated in the study completed the informed consent form correctly. The project was approved by the Ethics Committee of the Shanghai Mental Health Center (approval No. 2020-55). The study was conducted according to the ethical principles set out in the World Medical Association’s (WMA) Declaration of Helsinki.

### 2.2 Image acquisition

MRI images were acquired for all subjects using a 3T scanner (MAGNETOM Verio; Siemens Healthineers, Erlangen, Germany) using a 32-channel head coil at the Shanghai Mental Center. A foam pad was put under the patient’s head to prevent head movement. Structural images were acquired using a whole-brain three-dimensional sagittal T1-weighted scan, with the following parameters: sagittal acquisition; repetition time/echo time: 2300 ms/2.96 ms; inversion time: 900ms; flip angle: 9°; field of view: 256×256 mm; resolution: 1 × 1 mm; slice thickness: 1 mm (isotropic voxel of 1 mm).

### 2.3 Image processing

A T1-weighted image performed visual quality control on artefacts, preprocessing by the standard Recon-all pipeline overview implanted in FreeSurfer v7.0. We used the automatic procedures of hippocampal subfield segmentation and volumetric measurements of participant T1 images developed by T1-weighted MRI. The volume of hippocampus was further pre-processed using the standard FreeSurfer recon-all pipeline (version 7.0) (https://surfer.nmr.mgh.harvard.edu/fswiki/HippocampalSubfieldsAndNucleiOfAmygdala) (33). The hippocampus is divided into twenty-two subregions: the hippocampus proper, the hippocampal head, the hippocampal tail, subiculum head and body, cornu ammonis 1 body and head, parasubiculum, presubiculum body and head, cornu ammonis 2/3 body and head (CA2/3-body, CA2/3 head), cornu ammonis 4 body and head (CA4-body, CA4 head), granule cell-molecular layer-dentate gyrus (GC-ML-DG) body and head (GC-ML-DG-body, GC-ML-DG-head), the molecular layer hippocampus body and head (molecular-layer-HP-body and head), hippocampal–amygdaloid transition area (HATA), fimbria. Before further analysis, the hippocampal volume was corrected relative to intracranial volume (ICV).

### 2.4 Statistical analyses

IBM SPSS statistics for Windows, Version 19.0 (Armonk, NY, USA) was used for analysis of demographic and volume of subregions of the hippocampus.

We built a general linear model (one-way analysis of covariance) approach with the following variables: 42 hippocampal subfields and two total hippocampal volumes as dependent variables, and the diagnosis of MDD, BD-II, HC as an independent variable; the variables of age, sex, and total intracranial cavity volume (TICV) were covariates. We performed the pair-wise comparisons of hippocampal subfield volumes between MDD and HC, BD-II and MDD, MDD and BD-II with *post hoc* for primary analysis. The Bonferroni correction for this analysis and *post-hoc* pair-wise comparisons was applied to minimize type-1 error risk (P <0.05/44 = 0.001136). For demographic and clinical characteristics, we used an independent samples t-test to get the difference of HDRS, HCL-32 and family salary between MDD and BD-II. We applied the chi-squared tests on the distribution of sex of the MDD and BD-II group.

## 3 Results

### 3.1 Demographic data and characteristics

A total of 91 subjects (25 subjects with MDD, 36 with BD-II and 30 with healthy controls) was recruited to this study. Information regarding the sex, age, and other demographic features of subjects is given in **Table 1**. There were no significant differences in age (*P* = 0.052) or gender (*P* = 0.117) between MDD and BD-II. However, there was a significant difference in depressive symptom scores (P = 0.019) and HCL-32 scores (p = <0.001) between MDD and BD-II (**Table 1**).

**Table 1:** Demographic information for all participants.

		N	Mean	Standard deviation	t	P-value
HAMD	MD	25	35.440	10.508	2.416	0.019
	BD II	36	28.583	11.165		
HCL-32	MD	25	9.960	2.879		<0.001
	BD II	36	23.056	6.155		
Age	MD	25	25.240	5.532	-1.984	0.052
	BD II	36	28.610	7.129		

P-values for age, HAMD, and HCL-32 scores were obtained using an independent t-test.

BD: bipolar disorder; MDD: major depressive disorder; Ham-D: 24 item Hamilton Depression Scale; HCL-32: Hypomania Check List.

### 3.2 Hippocampal subfield volume differences between BD-II and healthy controls

**Table 2** lists the regions we examined and shows results for comparisons between healthy controls and patients with BD-II. We tested 22 regions on the left side and the right side, as well as the total volume of the hippocampus. Although many of these measurements are correlated, we decided to treat each test as an independent analysis and thus set a Bonferroni corrected 5% significance threshold of P = < 0.001 (0.05/44). We found that 2 results exceeded this threshold including the left HATA and right subiculum body.

**Table 2:** Hippocampal Differences between MDD,BD-II and healthy controls.

	All groups		BD vs HC		MDD vs HC		MDD vs BPD	
	F	P	t	P	t	P	t	P
Hippocampal tail L	0.807	0.45	1.178	0.243	0.916	0.364	0.134	0.894
Subiculum body L	3.543	0.033	1.809	0.075	2.69	0.01	-0.843	0.403
CA1 body L	5.791	0.004	2.606	0.011	3.097	0.003	-0.73	0.468
Subiculum head L	0.773	0.465	-0.093	0.926	1.04	0.303	-1.069	0.289
**Hippocampal fissure L**	**7.733**	**0.001**	**2.475**	**0.016**	**4.657**	**<0.001**	**-1.546**	**0.127**
Presubiculum head L	3.869	0.025	0.587	0.559	2.479	0.018	-2.057	0.044
CA1 head L	2.154	0.122	0.401	0.69	2.083	0.042	-1.551	0.126
Presubiculum body L	1.283	0.282	0.291	0.772	1.535	0.131	-1.332	0.188
Parasubiculum L	1.903	0.155	1.417	0.161	1.86	0.068	-0.643	0.522
Molecular layer HP head L	1.138	0.325	0.372	0.711	1.555	0.126	-1.08	0.284
Molecular layer HP body L	5.548	0.005	2.515	0.014	3.168	0.003	-0.67	0.505
GC ML DG head L	1.714	0.186	1.522	0.133	1.753	0.085	-0.284	0.778
CA3 body L	3.045	0.053	2.191	0.032	1.819	0.075	0.651	0.518
GC ML DG body L	2.207	0.116	1.96	0.054	1.685	0.098	-0.134	0.894
CA4 head L	1.819	0.168	1.499	0.139	1.806	0.077	-0.438	0.663
CA4 body L	1.788	0.173	1.771	0.081	1.469	0.148	0.068	0.946
Fimbria L	1.28	0.283	0.101	0.92	1.639	0.107	-1.316	0.193
CA3 head L	2.368	0.1	1.909	0.061	1.956	0.056	-0.055	0.956
**HATA L**	**14.616**	**<0.001**	**4.116**	**1.21E-04**	**5.948**	**<0.001**	**-1.526**	**0.132**
Whole hippocampal body L	5.167	0.008	2.214	0.03	3.167	0.003	-1.019	0.312
Whole hippocampal head L	2.496	0.088	1.008	0.317	2.413	0.019	-1.297	0.2
Whole hippocampus L	3.39	0.038	1.632	0.108	2.666	0.01	-1.066	0.291
Hippocampal tail R	1.543	0.219	1.434	0.156	1.472	0.147	-0.216	0.83
**Subiculum body R**	**9.296**	**<0.001**	**3.423**	**1.09E-03**	**4.455**	**<0.001**	**-0.564**	**0.575**
CA1 body R	2.605	0.08	1.763	0.083	1.987	0.052	-0.614	0.542
Subiculum head R	1.346	0.266	-0.577	0.566	1.003	0.32	-1.606	0.114
Hippocampalfissure R	2.07	0.132	0.257	0.798	2.062	0.044	-1.6	0.115
Presubiculum head R	2.341	0.102	0.985	0.328	2.08	0.042	-1.306	0.197
CA1 head R	0.683	0.508	0.646	0.52	1.136	0.261	-0.615	0.541
Presubiculum body R	2.419	0.095	0.683	0.497	2.113	0.039	-1.58	0.119
Parasubiculum R	5.585	0.005	2.661	0.01	3.301	0.002	-1.032	0.306
Molecular layer HP head R	1.04	0.358	0.481	0.632	1.367	0.177	-1.019	0.312
Molecular layer HP body R	3.758	0.027	2.021	0.047	2.563	0.013	-0.737	0.464
GC ML DG head R	1.376	0.258	1.048	0.299	1.647	0.106	-0.708	0.482
CA3 body R	0.256	0.775	0.685	0.496	0.541	0.59	0.042	0.966
GC ML DG body R	0.95	0.391	1.071	0.288	1.24	0.22	-0.386	0.701
CA4 head R	1.357	0.263	1.137	0.26	1.621	0.111	-0.589	0.558
CA4 body R	0.29	0.749	0.37	0.713	0.744	0.46	-0.446	0.657
Fimbria R	1.11	0.334	1.665	0.101	0.86	0.394	0.417	0.678
CA3 head R	1.868	0.16	1.338	0.186	1.893	0.064	-0.727	0.47
HATA R	2.904	0.06	1.489	0.142	2.436	0.018	-1.073	0.288
Wholehippocampal body R	4.724	0.011	2.267	0.027	2.976	0.004	-0.799	0.427
Whole hippocampal head R	1.695	0.19	0.868	0.389	1.822	0.074	-1.086	0.282
Whole hippocampus R	2.993	0.055	1.626	0.109	2.371	0.021	-0.935	0.353
Hippocampal tail L	0.807	0.45	1.178	0.243	0.916	0.364	0.134	0.894
Subiculum body L	3.543	0.033	1.809	0.075	2.69	0.01	-0.843	0.403

Bonferroni correction was applied: P <0.05/44 = 0.001136.

Significant hippocampal subfield volume differences appear in bold.

MDD, major depressive disorder; BD, bipolar disorder; HC, healthy controls; CA, cornu ammonis.

### 3.3 Hippocampal subfield volume differences between MDD and healthy controls

**Figure 1:**
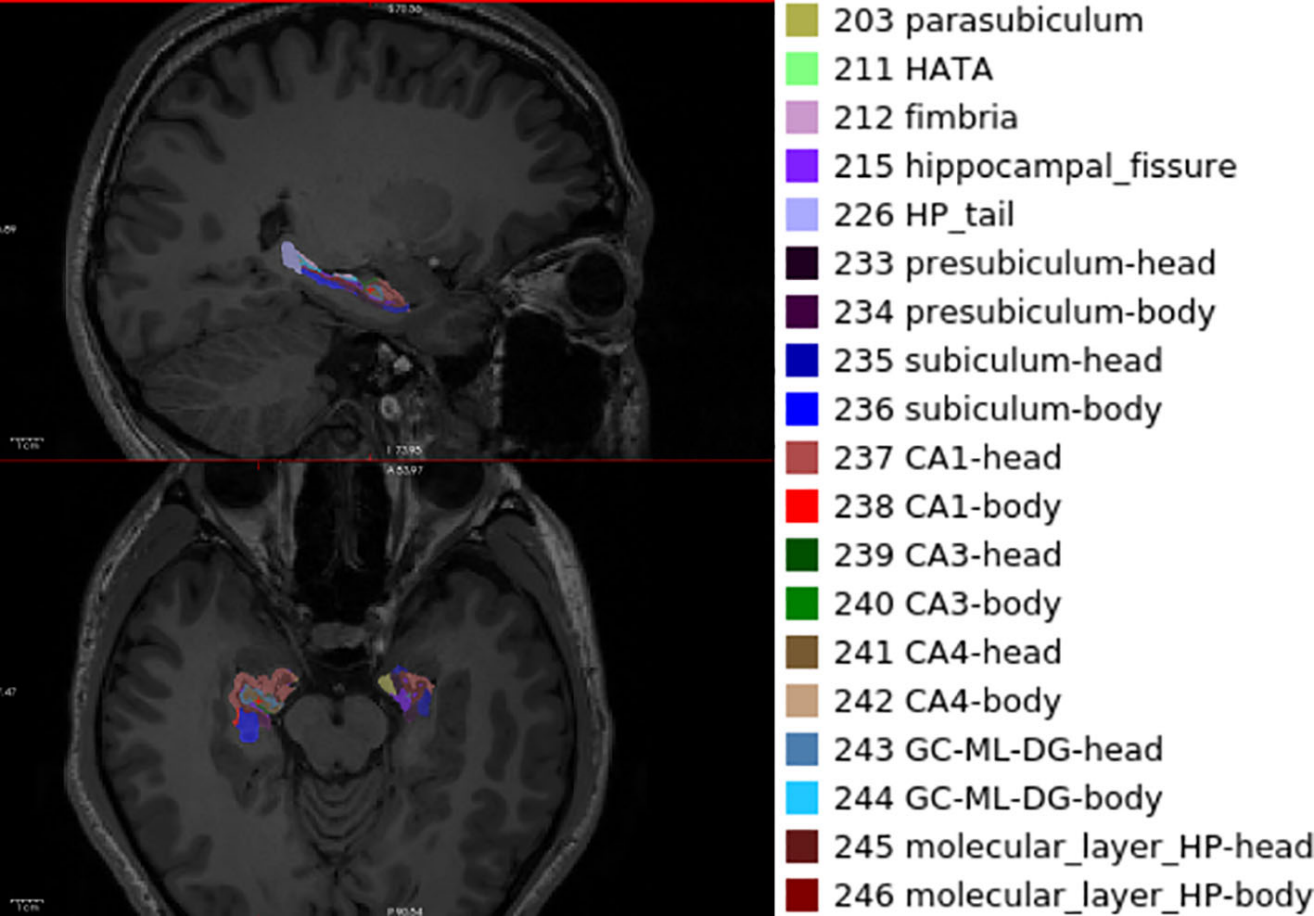
Illustration of hippocampal subfield segmentation by FreeSurfer V7.0.

**Table 2** shows results of comparisons between patients with MDD and HC. Since the measurements are the same as those collected for the analysis of BD-II, we applied the same significance thresholds. We found 3 results that exceeded the Bonferroni corrected threshold. We observed a significantly larger volume of the left hippocampal fissure and left HATA and the right subiculum body in MDD compared with the HC (**Figure 1**).

### 3.4 Hippocampal subfield volume differences between MDD and BD-II

We found no significant differences in any hippocampal measure between MDD, and BD-II.

## 4 Discussion

### 4.1 Summary of main findings in this study

We studied the difference of the hippocampal substructure between MDD and BD and HC, and compared the hippocampal substructure between MDD and BD-II. We found that a) in theBD-II, the hippocampal volume of the left HATA and right subiculum body was significantly increased. b) In MDD, the volume of the left hippocampal fissure, left HATA and right subiculum-body increased significantly. c) We found no significant difference in hippocampal substructure between MDD and BD-II.

### 4.2 Comparison with previous studies

When comparing the volume of the hippocampus in MDD to HC, most studies found that the volume is reduced (13, 17, 34, 35). We found that the volume of left HATA and right subiculum-body increased in patients with MDD (36). Previous studies have found that the left hippocampus is more reduced (36, 37), and we had similar findings showing that the left hippocampal body and the left HATA are most influenced. Yao found that subiculum and CA1 subregions of the bilateral hippocampus are prone to atrophy (17). Some researchers found a reduction in the volume of the hippocampal tail bilaterally, right hippocampal head and right hippocampus proper in MDD patients (14), showing that the right hippocampus is influenced too. However, most studies included patients with long term depression, or who were in remission. Previous studies have found that the volume of the left hippocampal protrusion decreased after the first or repeated episodes of MDD (13). Few studies involved the first episode of MDD with drug naïve patients. We found that an increase in the left hippocampal fissure, left HATA and right subiculum-body of the hippocampus might be characteristic of early-stage depression.

For BD-II, in this study we found that more subfields of the hippocampus are influenced from the left side to the right side, including the left HATA and right subiculum body, findings which are similar to previous studies. Some researchers found that the most affected sizes were in volume differences between BD-II and HC in the molecular layer, the hippocampal tail, cornu ammonis (CA4), and cornu ammonis (CA1) (11, 19). There are specific changes in subregions of the hippocampus in depressive episodes of bipolar disorder, such as cornu ammonis 1 (CA1), cornu ammonis 4 (CA4) (11, 30), the granule cell layer (GCL), molecular layer (ML), subiculum (sub). However, one study found that the volume of these subregions was increased, perhaps because of confounders such as medication, alcohol and illicit substance use, illness stage and the age of onset (19). Cao et al., recruited BD-I and BD-II disorder patients who were receiving treatment (11). They found that patients with BD (including BD-I and BD-II), had reduced volumes of hippocampal subfields, specifically in the left CA4, GCL, ML and both sides of the hippocampal tail, compared with healthy subjects. Another study recruited adolescent BD patients with adult BD, and found no reduction in the size of the hippocampus (38). They recruited subjects who were mainly young people with BD-II. Although some researchers found that BD-I has a severer reduction in hippocampal subfields than BD-II (11, 30), we still need to pay attention to the confounding effects on the hippocampus of the disease episode, progression and medicine treatment. Our study found that BD-II produced an increase in the volume of left HATA and right subiculum body of the hippocampal subregions in the early stage particularly with drug-naïve and young patient groups.

We did not find any difference between MDD and bipolar disorder. Cao found that the hippocampal subfields were more affected in BD-I compared with BD-II and MDD (11). Kyu-Man Han et al., found similar results and showed that no significant volume differences were observed between MDD and BD (26). Kyu-Man Han’s study only recruited subjects who were euthymic or in a depressive state. Another difference is that their study was conducted on patients with BD including BD-I and BD-II.who were already taking medicine (26). BD-I and BD-II may have different effects on the volume change of the hippocampus, so we should treat them differently. It is possible that the type of bipolar disorder, the effects of medicine, the episode and duration of the illness, and the number of episodes may affect the size of the hippocampus (11). In future, it will be necessary to compare the differences in the hippocampus in the early, middle and multiple episodes of BD-II seen in this study.

### 4.3 Implications

Although we found no significant difference between BD-II and MDD in the hippocampal subregions, there were more extensive changes on the left side in MDD. One implication is that there is more extensive cognitive impairment during the onset of MDD, such as decreased working memory (39, 40) and episode memory. Some studies show that the cognitive dimension of MDD is more extensive.

This study only compares the symptoms of MDD and BD-II, and in doing so it found significant differences. It attempts to explore the differences in symptom-related hippocampal subregions. However, no significant difference was seen in the subregion of the hippocampus between the two diseases. Analysis of the results suggests that: the sample size is relatively small, and that we need to expand the sample to explore whether there is a linear relationship between the more serious depressive symptoms and the smaller hippocampal volume in MDD. In BD-II, there is no such linear relationship.

Our study recruited subjects with the first onset of depression and BD-II, and our findings suggest that the increase of hippocampal volume may be an early pathological change. Many studies are based on the hippocampal contractile changes of recurrent or mixed episodes of bipolar disorder (11, 26). Our study suggests that changes in hippocampal enlargement may be related to inflammatory response (41, 42) in the early stage of the disease. Moreover, the inflammatory response of MDD may be more obvious, which needs more basic research to see whether this is so.

### 4.4 Limitations

Our study had the following limitations: 1) our power to detect an effect is limited by our small sample size. In a recent large meta-analysis of imaging data from patients with MDD, Schmaal et al. (43) estimated that 545 subjects per group would be needed to provide 80% power to detect difference in hippocampal volume at P-value=0.05. At this point we can only caution that ours is an exploratory study, generating hypotheses for further investigation.2) Our subjects were not matched for gender; Bipolar I is more common in men, while BD is more common in women (44). 3) Mixed episodes or rapid cycling of bipolar disorder is more likely to increase the risk of suicide, and such episodes cannot be evaluated. In this study, we did not assess whether patients with bipolar disorder had more frequent episodes or mixed episodes of BD, and which kind of clinical characteristics of bipolar disorder II were more likely to develop mixed episodes. 4) This study is cross-sectional. Only 20% of patients with bipolar disorder depressive episodes were diagnosed with bipolar disorder in the first year, and the diagnosis was often delayed for 5-10 years (45). This is possible because a diagnosis of BD is difficult to make early in the course of the disorder. In this study, the patients with first-episode MDD before the age of 30 could not be ruled out from BD-II. It will be necessary to conduct follow-up studies on patients with MDD to see if they develop BD in the next 5-10 years. 5) Hippocampal volume has a close relationship with cognition. This study did not include level of education as a covariate, and follow-up studies need to comprehensively assess the impact of this. 6) The age of onset and the prolonged duration of the disorder are not included the current study and should be discussed in future studies as influencing factors.7) the diagnostic system in this study used DSM-IV, and it should be updated in the future study and the related psychotherapy situation could be recorded when the participants were interviewed. 8) A limitation of this study is that it examined only the hippocampus. Future work should study more extensively the brain regions involved in regulating emotional stability.

## 5 Conclusion

From the data in this study, it can be concluded that there is no significant difference in subregions of the hippocampus between BD-II and MDD in the early development of BD-II. In the early stage of MDD, the volume of the hippocampal subregions including the left hippocampal fissure, left HATA and right subiculum-body regions are increased, possibly influencing working memory and episodic memory.

## Data availability statement

The raw data supporting the conclusions of this article will be made available by the authors, without undue reservation.

## Ethics statement

The studies involving humans were approved by the Ethics Committee of Shanghai Mental Health Center (batch number: 2020-55). The studies were conducted in accordance with the local legislation and institutional requirements. The participants provided their written informed consent to participate in this study. Written informed consent was obtained from the individual(s) for the publication of any potentially identifiable images or data included in this article.

## Author contributions

EC: Funding acquisition, Project administration, Resources, Writing – original draft. YZ: Data curation, Investigation, Methodology, Writing – review & editing. MW: Data curation, Investigation, Writing – review & editing. HC: Data curation, Investigation, Writing – review & editing. YC: Data curation, Investigation, Writing – review & editing. ZL: Data curation, Investigation, Writing – review & editing. YW: Data curation, Investigation, Writing – review & editing. HW: Data curation, Investigation, Writing – review & editing. YH: Data curation, Investigation, Writing – review & editing. HZ: Data curation, Investigation, Writing – review & editing. YL: Data curation, Investigation, Writing – review & editing. XL: Data curation, Investigation, Writing – review & editing. PZ: Data curation, Investigation, Writing – review & editing. WL: Data curation, Investigation, Writing – review & editing. YX: Project administration, Supervision, Writing – review & editing. YW: Project administration, Supervision, Writing – review & editing.
